# COVID-19 Vaccine Hesitancy in Healthcare Workers Amidst the Second Wave of the Pandemic in India: A Single Centre Study

**DOI:** 10.7759/cureus.17370

**Published:** 2021-08-22

**Authors:** Amey Joshi, Mallika Sridhar, Venkata Jaya Divya Tenneti, Vamana Devi, Sangeetha K T, Aditya B Nallaperumal

**Affiliations:** 1 General Medicine, Manipal Hospital, Bangalore, IND; 2 Microbiology, Manipal Hospital, Bangalore, IND; 3 Administration, Manipal Hospital, Bangalore, IND

**Keywords:** covid 19, vaccine, healthcare workers, sars-cov-2 vaccines, vaccine hesitancy

## Abstract

Introduction

Coronavirus disease 2019 (COVID-19) vaccine hesitancy amongst healthcare workers (HCW) has been reported in varying degrees in different parts of the world. In this study, we investigate the degree of vaccine hesitancy amongst HCWs and factors associated with it during the second wave of the pandemic in our centre.

Methods

We undertook this single-centre, cross-sectional study in an urban tertiary care hospital, using a modified Oxford COVID-19 vaccine hesitancy scale. We performed descriptive and appropriate univariate analysis. We used the Kruskal Wallis test as appropriate, and Spearman rank correlation to evaluate the relation between general attitude to vaccination and COVID vaccine hesitancy score.

Results

We obtained 223 responses. The majority of HCWs in our sample (n = 201; 90.1%) had received at least one dose of the vaccine. The mean (SD) Oxford vaccine hesitancy score was 28.54 ± 2.05, with no significant difference observed between doctors (28.45 ± 2.26) and nurses (28.68 ± 1.70), or across different specialities. Of the respondents, 92.7% (n = 216) responded positively to taking the vaccine. The lack of dependents at home was the only significant contributor to vaccine hesitancy. Age, gender, marital status, and COVID-19 infection status did not significantly affect vaccine hesitancy.

Conclusion

We found a significantly lower degree of hesitancy towards COVID-19 amongst HCWs in our centre during the pandemic’s second wave. A more comprehensive and multi-centric study is required to validate this finding.

## Introduction

January 16th, 2021 marked the first day of India's vaccination efforts against coronavirus disease 2019 (COVID-19). Within just 24 days, it became the fastest country to vaccinate six million of its population [1]. Despite the initial success of the "world's largest vaccination program", the low supply of vaccines and emerging doubts regarding vaccine efficacy and side effects have slowed down vaccine acceptance [2,3]. As of late 2020, vaccine acceptance rates worldwide ranged from 23.6% to 97% in the general population and 27.7% to 78.1% in healthcare workers (HCW) [2]. During the first wave of the pandemic in India, the vaccine acceptance rate amongst HCWs in India, who were among the first to receive the vaccine, was 79.3% [4]. Their acceptance of the vaccine is crucial to ensure primary prevention of the disease and the appropriate dissemination of vaccine-related information.

The second wave of the pandemic began mid-March 2021 and peaked in early May 2021 [5]. This wave set multiple new records in terms of overall incidence and mortality [5]. This instilled fresh fear throughout the nation as the population scurried to receive available vaccines. This pilot study evaluated vaccine acceptance rates among HCWs in an urban tertiary referral centre during the second wave of the COVID-19 pandemic.

## Materials and methods

We conducted this prospective cross-sectional study between April 10th and June 10th, 2021, at a single tertiary care centre in Bangalore, India. After obtaining ethical approval from the Institutional Review Board, we distributed our questionnaire via the web-based Google Forms platform to HCWs (consultants, registrars, resident doctors, and nurses) working in COVID and non-COVID sections of the hospital. We took electronic consent from the participants before the administration of the questionnaire. The questionnaire comprised 20 items that focused on demographic details, attitude towards COVID 19 vaccine, exposure risk, and reasons for vaccine hesitance. Included in the 20 questions was a modified Oxford COVID-19 vaccine hesitancy scale comprised of six items, with each item graded based on the Likert scale (1-5) [6]. The question of "If the COVID 19 vaccine were available at the local pharmacy, I would" included in the original Oxford vaccine hesitancy scale was excluded from the form due to the lack of such a provision in India. The highest possible score in the currently employed questionnaire was 30, and the lowest was 6, 30 being the most compliant and 6 being the most hesitant to vaccine acceptance.

We analysed the data using Statistical Package for Social Sciences (SPSS) 25.0 software (IBM SPSS Statistics, Armonk, NY, USA). Categorical data were presented as frequencies and proportions and compared using independent sample t-test or chi-square test as appropriate. Kruskal Wallis test compared independent variables with more than two responses to the primary outcome. Spearman rank correlation was performed to evaluate the relation between general attitude to vaccination and COVID vaccine hesitancy score. We considered a p-value of less than 0.05 statistically significant.

## Results

Out of 234 HCWs, 223 consented to our survey. Of the 223 respondents, 60.9% (n = 136) were registered doctors, and 39.01% (n = 87) were registered nurses. The demographic details of the participants are presented in Table 1. The majority of the participants (90.1%; n = 201) had received at least one dose of the vaccine, and 82.1% (n = 183) had received both doses of the vaccine. The majority of the participants received the Oxford-Astrazeneca (COVISHIELD) vaccine (n = 192, 95.5%), while the remaining received the Bharath Biotech (COVAXIN).

**Table 1:** Demographic details of HCWs and significance with vaccine hesitancy. *Paraclinical departments include microbiology, pathology, and psychiatry. HCWs - healthcare workers; COVID-19 - coronavirus disease 2019.

Variable	N (%) (Total = 223)	P-value
Age (years)		
<34	112 (50.2)	0.07
≥35	111 (49.8)	
Gender		
Male	85 (38.1)	0.24
Female	138 (61.9)	
Profession		
Doctors	136 (60.9)	0.41
Nurses	87 (39.1)	
Department		
Surgery	46 (20.6)	0.91
Medicine	49 (22)	
Critical care	24 (13)	
Paraclinical*	10 (4.5)	
Nursing	87 (36.8)	
Not disclosed	7 (3.1)	
Role		
Consultant	82 (36.8)	0.09
Registrar	24 (10.8)	
Training doctors	30 (13.5)	
Nursing	87 (39)	
Marital status		
Married	151 (67.7)	0.053
Single	72 (32.3)	
Children/elderly/dependents at home		
Yes	149 (66.8)	0.002
No	74 (33.2)	
Have you had COVID-19		
Yes, lab-confirmed	40 (17.93)	0.19
No, lab-confirmed	72 (32.28)	
I may have had it, did not get tested	21 (9.4)	
Did not have it, but have not got tested	90 (40.3)	
Have you taken the COVID-19 vaccine?		
Yes	201	0.004
No	22	
Vaccine received		
Oxford-Astrazeneca (Covishield)	192 (86.1)	
Bharath BioTech (Covaxin)	9 (4)	
Not taken	22 (9.9)	

**Figure 1:**
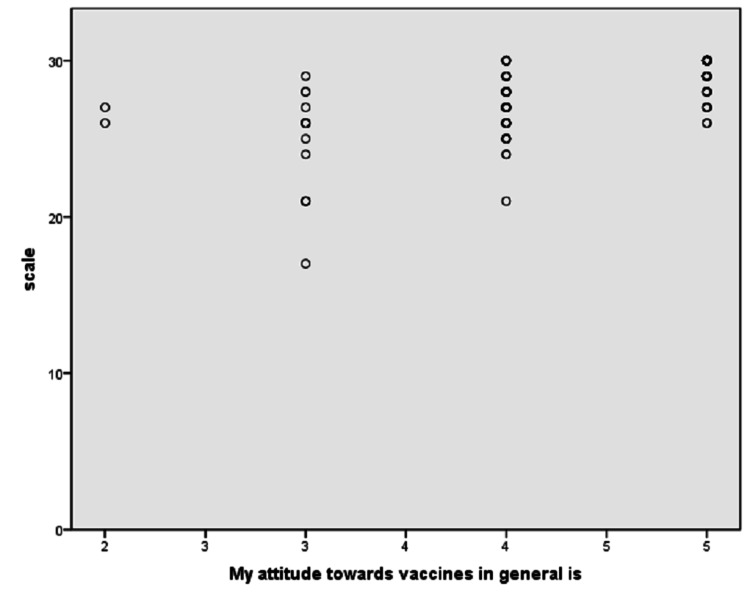
Correlation of COVID-19 vaccine acceptance and the general opinion of vaccines.

Reasons for vaccine hesitancy among HCWs were insufficient information regarding the vaccine (n = 1; 0.4%), fear of unknown adverse effects (n = 1; 0.4%), doubt in vaccine effectiveness (n = 1; 0.4%), distrust in vaccine company (n = 2; 0.8%), and fear of vaccine's effect on current pregnancy (n = 1; 0.4%). Of the respondents, 216 (92.7%) responded positively about taking the vaccine. Demographics including age, gender, marital status, role in the hospital, and field of practice were not significantly associated with vaccine acceptance. Having dependents at home, including children or the elderly, significantly contributed to vaccine acceptance when the two groups were compared with the Oxford vaccine hesitancy scale using an independent t-test (28.83 ± 1.86 vs 27.95 ± 2.31; p-value = 0.002). Doctors and nurses scored similarly in the vaccine hesitancy scale (28.45 ± 2.26 vs 28.68 ± 1.70; p-value=0.41). We found a moderately positive correlation between the general attitude towards vaccine acceptance (graded based on the Likert scale 1-5; 1 - least compliant and 5 - most compliant) and COVID-19 vaccine acceptance by Spearman rank correlation analysis (ρ = 0.75, p-value <0.001) (Figure 1).

Due to the limited number of responses for vaccine hesitancy or uncertainty (n = 4 and n = 3, respectively), we did not compare the average vaccine hesitancy score to those who responded positively to the vaccine.

## Discussion

Our study showed that the COVID-19 vaccine hesitancy rate in HCWs during the second wave of the pandemic was 6.3%. This was significantly lower than vaccine hesitancy rates in HCWs before the second wave in India, estimated to be 20.7% [4]. At this point, vaccine safety, efficacy, and possible side effects were the top reasons for vaccine hesitancy. Other reasons cited included insufficient knowledge or distrust regarding the vaccine, its rapid development and those responsible for it, and the spread of misinformation on social media [7]. Some of these concerns, although reported in our study, were less prevalent. Vaccine hesitancy due to its effect on active pregnancy was also a reported concern in the present study. At the time of writing, on July 2nd, 2021, the Union Health Ministry of India, on recommendations from the National Technical Advisory Group on Immunization (NTAGI), announced the safe use of the COVID-19 vaccine even in pregnancy, addressing this concern.

Previous studies revealed greater reluctance among HCWs working in low-risk settings such as out-patient departments and outreach service teams [8,9]. In contrast, we observed a uniform vaccine acceptance score throughout all medical specialties. Further, prior studies [7] found that gender and older age were not significant contributors to vaccine hesitance. At the same time, the presence of dependents at home was associated with lower hesitancy.

We speculate that the primary reason for greater vaccine acceptance in our sample is the unprecedented upsurge of new cases and the resultant death toll in this wave of the pandemic. Furthermore, the recent availability of phase three results of the Bharat Biotech (COVAXIN) vaccine in March 2021 [10] and the availability of more data regarding the side effects and efficacy of the other available vaccinations - Oxford/AstraZeneca COVID-19 AZD1222 (Covishield) and Sputnik V vaccine - may have increased vaccine acceptance.

As India gears up for a potential third wave of the COVID-19 pandemic and ramps up vaccine production, it is imperative to ensure nationwide vaccine acceptance. The present study is limited due to its pilot approach in an urban tertiary referral centre. As a result, the findings may not accurately represent the wider HCW population. A more comprehensive analysis is necessary to confirm the extent of COVID-19 vaccine acceptance in HCWs. As of July 20th, 2021, a dismal 6.3% of India's 1.38 billion people have been vaccinated against COVID-19 [5]. As more information and data regarding the COVID-19 vaccines have become available, many previous concerns of vaccine adverse effects, reliability, and efficacy have been tackled. A vaccine acceptance of 93.7% in HCWs in the current study is highly encouraging and is a testament to this. Vaccination awareness campaigns led by HCWs supported by the government in areas of high vaccine reluctance can help speed up vaccine uptake and mitigate a potential next wave of the pandemic.

## Conclusions

We found a significantly lower vaccine hesitancy towards COVID-19 amongst HCWs in our centre during the second pandemic wave. A more comprehensive and multi-centric study is required to validate this finding.
